# Comparative Analysis of Serum Magnesium Ion Levels Using Three Measurement Methods: Spectrophotometry, Atomic Absorption Spectrophotometry, and Inductively Coupled Plasma With Optical Emission Spectrophotometry

**DOI:** 10.1155/ianc/8853568

**Published:** 2025-03-25

**Authors:** Raja Iqbal Mulya Harahap, Intanri Kurniati, Nida Suraya, Tiene Rostini, Bejo Ropii, Maulidwina Bethasari

**Affiliations:** ^1^Department of Clinical Pathology, Faculty of Medicine, Universitas Padjadjaran, Bandung, West Java, Indonesia; ^2^Faculty of Medicine, Universitas Padjadjaran, Bandung, West Java, Indonesia; ^3^Department of Clinical Pathology, Faculty of Medicine, University of Lampung, Bandar Lampung, Indonesia; ^4^Department of Clinical Application and Research, Preanger Institute for Biomedical Engineering, Science, and Technology, Bandung, West Java, Indonesia; ^5^Department of Pharmacy, Faculty of Science and Technology, Universitas Muhammadiyah Bandung, Bandung, West Java, Indonesia

**Keywords:** ions, magnesium, serum, spectrophotometry

## Abstract

Magnesium is a cation that plays as an important cofactor in various enzymatic reactions. It is the fourth most abundant cation in the body after sodium, potassium, and calcium. There are various magnesium measurement methods available such as spectrophotometry, atomic absorption spectrophotometry, and inductively coupled plasma–optical emission spectrophotometry. These measurement methods have various advantages and disadvantages in measuring magnesium levels in serum. This study aimed to compare the magnesium measurement results by using three different methods. A total of 221 samples were examined for magnesium levels using spectrophotometry, atomic absorption spectrophotometry, and inductively coupled plasma–optical emission spectrophotometry methods. The results were then grouped into hypomagnesemia, noromagnesemia, and hypermagnesemia according to normal values. The mean and standard deviation were calculated and compared across three different methods. The mean and standard deviation of serum magnesium ion levels measured using spectrophotometry, atomic absorption spectrophotometry, and inductively coupled plasma–optical emission spectrophotometry methods were 1.84 ± 0.43, 1.86 ± 0.43, and 1.85 ± 0.43 (mg/dL), respectively. There were no significant differences (*p* value > 0.05) in serum magnesium levels using spectrophotometry, atomic absorption spectrophotometry, and inductively coupled plasma–optical emission spectrophotometry measurement methods, indicating similar reliability among the methods.

## 1. Introduction

Magnesium (Mg) is the fourth most abundant mineral in the human body, is also the most abundant divalent cation intracellularly, and has an important role in various physiological functions. Mg deficiency can be caused by various factors, such as reduced intake, excretion disorders, and medical diseases which can cause changes in homeostasis and are often associated with various complaints. Due to its many and ubiquitous roles, magnesium status examination can be used as a pathological marker in acute and chronic conditions [1]. Various methods for measuring magnesium levels in vitro have been developed since the late 1970s, such as fluorescent probes and nuclear magnetic resonance (NMR) which were only able to check total magnesium levels including free form (ion) and protein-bound form [1]. Nowadays, current method such as electrode ion spectrophotometry, atomic absorption spectrophotometry (AAS), or inductively coupling plasma–open emission spectrophotometry (ICP-OES) enabling healthcare facility to measure magnesium ion levels specifically which has more physiological role compared to when bound to protein [1, 2].

The metabolism of Mg and its distribution in the body was initially carried out using radioactive Mg isotopes, but its use was very limited due to its short half-life and the large risk of radiation exposure to humans. These studies found that the half-life of Mg in the body is in the range of 41–181 days. This is in accordance with clinical observations which state that it takes approximately 3 months to restore normal magnesium levels after losing 20% magnesium [3].

Magnesium ion levels in the blood are greatly influenced by various factors, both preanalytic, analytical, and postanalytic factors. Blood samples must be prepared carefully to prevent contamination of magnesium with anticoagulant components, and its concentration is better measured with serum samples rather than plasma. Hemolysis can increase Mg concentration because erythrocytes contain higher Mg than serum. Other research shows that these preanalytic factors can be a significant source of error and influence the measurement of magnesium ion levels. Serum Mg ion levels also depend on dietary intake and intestinal absorption and kidney function. Kidney function, both filtration and reabsorption, plays a role in maintaining magnesium ion levels at normal levels.

Various other medical conditions that can affect magnesium ion levels include diabetes, obesity, chronic heart disease, critical illness requiring admission to an intensive unit, asthma, and Crohn's disease. Several types of drugs that are used routinely can also interfere with magnesium metabolism including digitalis, acetaminophen, insulin, magnesium supplements or preparations, and various other drugs [1, 4].

Currently, the method most widely used in laboratories for measuring the magnesium level is the spectrophotometric method; however, the AAS and ICP-OES methods can also be used in measuring magnesium, considering that both methods have lower measurement sensitivity and can be used in measurement panels for various types of trace minerals. There has been a lack of studies comparing the results of magnesium ion measurements using these three methods, particularly given the limited number of medical laboratories that utilize the AAS and ICP-OES methods [5–7]. This study aimed to compare the magnesium ion level measurement using three different methods (spectrophotometry, AAS, and ICP-OES) in healthy patients.

## 2. Methods

### 2.1. Materials

The study utilized several reagents and equipment for magnesium concentration measurement. Key reagents included concentrated hydrochloric acid (HCl), demineralized water, lanthanum chloride solution (LaCl_3_), pure metallic magnesium (Mg), and the Alinity C magnesium reagent kit. The equipment employed comprised an ICP–mass spectrometer (Difotek DF-AAS01), AAS (Shimadzu AA620), and ICP-OES Avio 550). Additionally, serum separating tubes (SSTs) were used for blood sample collection, along with a centrifuge for sample preparation. Transfer pipettes and tips facilitated accurate handling of liquids, and gloves ensured safety during the procedures. A digital display or printer was utilized for result reading, and calibration standards for magnesium were also prepared. An Alinity C analyzer (Abbott) was employed for the spectrophotometric measurements.

### 2.2. Population

Serum samples were collected from adult men and women aged > 18 years who underwent a Medical Check-Up at Hasan Sadikin Hospital and Abdoel Moeloek Hospital Lampung and were declared healthy by the doctor on duty at the Medical Check-Up. The exclusion criteria for this patient were subjects with chronic kidney disease, a history of using magnesium sulfate in the last 6 months, and a history of treatment in the intensive unit within the last 3 months. Subjects who met the inclusion and exclusion criteria were then asked for informed consent by the researcher. The research was conducted in Bandung in the period August–December 2022, originating from a HETI research grant, a collaboration between FK Lampung University and FK Padjadjaran University. Ethics approval was issued by the Research Ethics Committee of FK Lampung University and FK Padjadjaran University Bandung number 2000/UN.2618/PP.05.02.00/2022.

About 3–4 cc of blood was taken from the patients. After the blood was collected, it was centrifuged in the laboratory, then divided into three aliquots, each aliquot was used for magnesium examination using spectrophotometric, AAS, and ICP-OES methods. Samples were stored at −80°C until the number of samples was sufficient. Magnesium ion measurement was performed using the spectrophotometric method at the Hasan Sadikin Hospital Clinical Laboratory, while magnesium measurement using the AAS and ICP-OES methods were performed at the Central Laboratory of Padjadjaran University.

### 2.3. Serum Mg Measurement

#### 2.3.1. Spectrophotometry

The spectrophotometry procedure utilized in serum Mg measurement was based on Alinity C Magnesium reagent kit which is based on enzymatic reaction forming NADPH as a reaction product. Prepared samples were analyzed by using Alinity C (Abbott) according to the procedure on the kit manual.

#### 2.3.2. ICP-MS

• Principle of Examination
  In the ICP-MS system, serum samples are converted into aerosol form. The aerosol is then burned with argon and subsequently dissociated into individual atoms. The atomic charges of magnesium (Mg) that pass through the mass spectrometer are amplified by the instrument and converted into quantitative concentrations.
• Sample Preparation
  Whole blood samples in SST must be allowed to stand until complete blood clotting occurs. The samples are then centrifuged at a speed of 3000 rpm for 10 min to separate the serum from the blood cells. The samples in SST can be used directly for examination.
• Measurement
  The examination procedure begins with the insertion of the SST containing the serum sample into the ICP-MS instrument, which automatically aspirates the sample and dissociates it into individual atoms. Following this, the instrument amplifies the atomic charges of magnesium (Mg) to facilitate detection by the detector. For result reading, the ICP-MS instrument then converts the amplified atomic charges of Mg into quantitative serum magnesium concentrations, utilizing a previously established calibration curve to ensure accuracy in the measurements.

#### 2.3.3. Atomic Absorption Spectometric

• Preparation of Lanthanum Chloride Solution
  The preparation of lanthanum chloride solution (87 g/L) involved mixing 29 g of La_2_O_3_ with a few milliliters of demineralized water to form a slurry. Concentrated hydrochloric acid (250 mL, specific gravity 1.19) was then slowly added while stirring, with precautions taken due to the potential for violent reactions. The resulting solution was subsequently diluted to a final volume of 500 mL with demineralized water.
• Preparation of Magnesium Standard Solution
  For magnesium standard Solution I (1.00 mL = 0.50 mg Mg), 0.500 g of pure metallic magnesium was dissolved in a minimal amount of dilute hydrochloric acid and then diluted to 1000 mL with demineralized water. A series of at least six magnesium working standards were prepared, containing concentrations ranging from 0.01 to 5.0 mg/L or from 2.5 to 50 mg/L, through appropriate dilution of magnesium standard Solution I. In each case, 1.0 mL of LaCl_3_ solution was added for every 10 mL of working standard. Additionally, a demineralized water blank was prepared.
• Sample Analysis
  For sample analysis, 1.0 mL of lanthanum chloride solution was added to 10.0 mL of each sample solution. The blank was aspirated to establish the automatic zero control, and the automatic concentration control was calibrated using at least six standards. Instrument calibration was conducted for each set of samples analyzed and was checked at reasonable intervals.
• Determination of Magnesium Concentration
  The milligrams per liter of dissolved or total recoverable magnesium in each sample solution were determined from the digital display or printer during aspiration. For samples with magnesium concentrations exceeding the working range, appropriate dilution was performed, and the results were adjusted accordingly. The milligrams per liter of suspended recoverable magnesium were calculated by subtracting the dissolved magnesium concentration from the total recoverable magnesium concentration.
• Calculation for Bottom-Material Samples
  The milligrams per liter of magnesium in bottom-material samples were determined using the same methodology described above, with subsequent calculations conducted as required to ensure accurate reporting of results.

### 2.4. Statistical Analysis

Statistical analysis was carried out using a descriptive test to determine the description and characteristics of the research subjects, followed by a data normality test with the Kolmogorov–Smirnov test. ANOVA was performed if the data were normally distributed; otherwise, the Kruskal–Wallis test was used. Statistical analysis was carried out with SPSS Ver 21.0. The *p* value will be considered significant if < 0.057.

## 3. Results

Two hundred and twenty-one research subjects who met the inclusion and exclusion criteria were included in this study. The characteristic of the research subject is shown in Table 1. The majority of the research subjects were more than 40 years. The number of female and male participants were similar.

After the blood samples were collected, they were divided into three portions for the measurement of magnesium levels using spectrophotometry, AAS, or ICP-OES. The results of these measurements are presented in Table 2.

The Kolmogorov–Smirnov test showed that the data obtained from the spectrophotometry method were not normally distributed (*p* value < 0.05), while the data in the AAS and ICP OES groups were normally distributed (*p* values 0.2 and 0.055, respectively). Therefore, the Kruskal–Wallis test was carried out as an alternative test to ANOVA which cannot be used because of the non-normal distribution of the data. The results of the Kruskal–Wallis test showed that there was no significant difference between the average magnesium levels of the three types of test equipment studied (*p* value = 0.921). The results of this analysis can be seen in Table 3 below.

## 4. Discussion

From the results of this research, it was found that the most research subjects were in the age group > 60 years, followed by the age group 41–59 years, the numbers of which were not too different between the two groups, and the age group under 40 years had the least number. This was influenced by the demographics of visits to the medical check-up unit at the research location, which was more in the age group over 40 years. Based on gender, the number of research subjects between men and women was similar. This was also equally influenced by the demographics of the research subject's visits.

The mean and standard deviation (Table 2) for the three methods were similar. It can be seen sequentially that the mean and standard deviation values for the spectrophotometric, AAS, and ICP-OES methods were 1.84 ± 0.43, 1.86 ± 0.43, and 1.85 ± 0.43 (mEq/L), respectively. From the comparison test with Kruskal–Wallis, the *p* value between subject groups was 0.921, and this shows that there was no significant difference in serum magnesium ion levels from three methods. The categorization of normomagnesemia, hypomagnesemia, and hypermagnesemia was also similar for the three groups. This study showed that there was no difference between the three measurement methods in measuring serum Mg level in vitro as well as for the categorization of serum magnesium ion levels in research subjects.

In these three measurement methods, there were several differences and similarities in the principles of measuring metal content in samples. The AAS method has the principle of absorbing light by atoms, and the atoms absorb light at certain wavelengths, depending on the nature of the element. Light at this wavelength has enough energy to change the electronic energy level of an atom. Magnesium atoms in serum will be excited in higher orbitals in a short time by absorbing radiation energy at certain wavelengths. The difference in the absorbance value of the blank (without the target sample) compared to the test sample is the concentration value of the desired target substance. When the concentration value is known, other mass units can be found when converted. The measurement requires a standard curve whose elements are the concentration of the analyte compared to the absorbance value. A standard curve is created using a solution of known concentration of the substance to be tested with various concentration differences. The basis of this method is almost the same as the spectrophotometric method used in various routine chemical examinations in laboratories [7, 8].

The ICP-OES method is an instrument analysis technique used to analyze chemical elements in various matrices or samples. This instrument is an emission spectroscopy that uses ICP to produce excited atoms and ions that emit electromagnetic radiation with wavelengths specific to each element. Plasma comes from ionized gas (mostly argon) and is produced and retained by inductive coils. The temperature of the plasma source is in the range between 6000 and 10,000 K. The intensity of the emission rays produced by each element is directly proportional to its concentration [9].

The AAS and ICP-OES methods are used less frequently because the sample preparation step is more difficult because the sample must be destroyed with the help of nitric acid, and often the concentration of nitric acid that is not standardized between samples will affect the excitation and color changes that occur. In addition, these two methods require different standard matrices and blanks, so that there can be differences in the blank reference which causes a shift in the calibration curve and the risk of miscalculation of the analyte levels in the sample. However, these two methods have similarities with the widely used spectrophotometric method. They both measure analyte levels by comparing color changes to standards. These two methods also have other advantages, namely, that they have a detection limit that is much lower than the standard spectrophotometry, so that these two methods can be used to detect levels of other trace minerals that are not measured by spectrophotometric methods [8, 9].

There are very few other similar studies that compare the results of measuring magnesium with the three methods, another study from Yokokawa (2020) comparing zinc levels with the AAS and ICP-OES methods found that there was no significant difference in measuring the levels of the two analytes. Other research from Kannan (2018) also showed that there is no significant difference between metide AAS and ICP-OES in measuring chromium levels in the Type II diabetes mellitus patient population [10, 11]. The results of this research show that for measuring several types of metals, various methods can be used.

## 5. Conclusions and Suggestions

From this study, it was concluded that there were no significant differences in serum magnesium ion levels measured using the spectrophotometry, AAS, and ICP–open emission spectroscopy methods, so these three methods can be used as a method for examining serum magnesium ion levels in vitro. Other research with a larger sample size can be carried out to validate the method, and other research can also be carried out with specific groups of subjects so that it can be linked to the clinical condition of the research subjects.

## Figures and Tables

**Table 1:** Characteristics of research subjects (*n* = 221).

Characteristics		Number of *n* (%)
Age	≤ 40 years	12 (5.43%)
	41–59 years	104 (47.06%)
	≥ 60 years	105 (47.51%)

Gender	Man	107 (48.42%)
	Woman	114 (51.58%)

**Table 2:** Magnesium levels from three different measurement methods.

No	Method	Reference value (mg/dL)	Mean (±SD)	Population number (number [percentage]) based on magnesium levels (mEq/L)		
				Hypomagnesemia (< 1.7)	Normomagnesemia (1.7–2.3)	Hypermagnesemia (> 2.3)
1	Spectrophotometry	1.7–2.3	1.84 ± 0.43	73 (33.03%)	128 (57.92%)	20 (9.05%)
2	AAS	1.7–2.3	1.86 ± 0.43	75 (33.94%)	121 (54.75%)	25 (11.31%)
3	ICP-OES	1.7–2.3	1.85 ± 0.43	73 (33.03%)	124 (56.11%)	24 (10.86%)

**Table 3:** Differences in average values of magnesium levels using three different methods.

	Spectrophotometry	AAS	ICP-OES	*p* value
Mean ± standard deviation of magnesium ion levels (mg/dL)	1.84 ± 0.43	1.86 ± 0.43	1.85 ± 0.43	0.921

## Data Availability

The data that support the findings of this study are available from the corresponding author upon reasonable request.
